# Pretreatment gut microbiome composition as a pharmacomicrobiomic prognosticator of conventional and biologic DMARD response in rheumatoid arthritis: A critical narrative review

**DOI:** 10.1016/j.cirep.2026.200307

**Published:** 2026-08-19

**Authors:** Ali Saad Al-Budairi, Abdullah Salim Al-Karawi, Ali M. Atoom

**Affiliations:** a Branch of Biology, Department of Science, College of Basic Education, Wasit University, Kut, Iraq; b Department of Microbiology, College of Science, Mustansiriyah University, Baghdad, Iraq; c Department of Medical Laboratory Sciences, Faculty of Allied Medical Sciences, Al-Ahliyya Amman University, Amman, 19328, Jordan

**Keywords:** Gastrointestinal microbiome, Arthritis, rheumatoid, Antirheumatic agents, Biological products, Treatment outcome, Biomarkers, Drug resistance, Pharmacogenomics, Dysbiosis, Butyrates

## Abstract

**Background:**

Rheumatoid arthritis is a chronic inflammatory disease that is estimated to affect 17.6 million people worldwide. Even with the development of biologic therapies, 30–40% of patients do not respond to the first-line therapy and others lose their response to therapy over time. Current serological and clinical predictors show limited accuracy, highlighting the need for more reliable treatment-response biomarkers.

**Objective:**

To evaluate critically the published evidence linking pretreatment gut microbiota composition to subsequent response to conventional synthetic and biologic DMARDs in rheumatoid arthritis, to appraise the pharmaco microbiomic mechanisms through which the microbiome may modify drug action, and to map the key methodological challenges that restrict translation to clinical practice.

**Search strategy and methods:**

A structured narrative review was conducted with a reproducible bibliographic search of PubMed/MEDLINE, Scopus, Web of Science, Cochrane Library, and DOAJ covered January 2015–April 2025. After deduplication and two-reviewer screening, 55 studies were included. Due to methodological heterogeneity, findings were synthesized narratively rather than by meta-analysis.

**Key findings:**

Pretreatment abundance of butyrate-producing Lachnospiraceae principally Subdoligranulum (AUC = 0.669) and Fusicatenibacter (AUC = 0.665)—was reproducibly associated with favourable csDMARD outcomes at six months. A composite gut microbiome-based model achieved AUC = 0.65 in independent validation (IMRABIOME cohort), which exceeded models relying exclusively on standard clinical variables (approximately AUC = 0.61). Prevotella copri was found in 75% of newly diagnosed RA patients versus 21.4% of healthy controls (p < 0.001). Shannon alpha-diversity was substantially reduced across RA cohorts, with a pooled SMD of −0.61 (95% CI: −0.89 to −0.33) relative to healthy controls. Pharmacomicrobiomic experiments confirmed direct microbial catabolism of methotrexate by pretreatment faecal microbiota, establishing a plausible causal link between baseline microbiome composition and drug bioavailability.

**Conclusion:**

Pretreatment gut microbiome composition may predict RA treatment response, but clinical translation requires large multicenter validation, standardized sequencing and bioinformatics, and ethnically diverse cohorts.

## Highlights

• Pretreatment gut microbiome composition predicts DMARD response in treatment-naive RA.
• Subdoligranulum (AUC 0.669) and Fusicatenibacter (AUC 0.665) are top microbiome predictors.
• Composite microbiome models (AUC 0.65) outperform clinical biomarker models (AUC ∼0.61).
• Gut bacteria directly catabolize methotrexate, influencing drug bioavailability and response.
• Methodological harmonization and ethnically diverse cohorts are essential for clinical translation.

**What this review adds**

Numerous narrative and systematic reviews have documented gut microbial dysbiosis in rheumatoid arthritis, yet three substantive aspects of the current literature have remained underexplored. This review addresses each in turn.

First, whereas prior publications have generally catalogued cross-sectional dysbiosis, the present work restricts its evidence synthesis to pretreatment microbiome composition as a predictor of pharmacological response in treatment-naïve patients. This framing is clinically more actionable, since it positions the microbiome as an instrument for prospective treatment selection rather than as a retrospective disease marker.

Second, this review integrates pharmacomicrobiomic mechanisms—particularly the experimentally confirmed direct catabolism of methotrexate by pretreatment gut bacteria—with biomarker prediction data. This integration supports the argument that the microbiome is not merely a correlational bystander but may actively shape drug bioavailability and thereby determine response magnitude.

Third, the review provides a structured performance appraisal of published prediction models, benchmarking current AUC values against the proposed clinical actionability threshold of 0.70. This analysis explicitly quantifies the performance shortfall that must be bridged before microbiome-based biomarkers can support clinical decision-making, and it maps a concrete methodological reform agenda targeted at closing that gap.

Alt-text: Unlabelled box dummy alt text

## Introduction

Rheumatoid arthritis is one of the more common inflammatory arthritides, and among the more damaging. Persistent inflammation of the synovium drives destruction of bone around the joints, and many patients also develop problems outside the joints that erode both function and daily quality of life. The 2019 Global Burden of Disease study estimated that 17.6 million people live with the disease, with about 13.5 new cases per 100,000 person-years each year and women affected roughly two to three times as often as men [1,2]. The economic toll is heavy as well. Patients whose disease stays active often leave work early, lose productivity, and run up considerable medical costs [3].

Treatment has changed dramatically over the last twenty years. One by one, biologic agents have entered practice: drugs against TNF-alpha, against the interleukin-6 receptor, against T-cell co-stimulation and B-cell surface markers, and, more recently, small molecules that block intracellular JAK signalling. In biologic-naïve patients, Phase III trials of TNF inhibitors report ACR20 responses of 58 to 65% by week 24 [4], and IL-6 blockade performs at least as well, with a particular edge in seronegative disease [5]. Yet a stubborn problem remains. Somewhere between 30 and 40% of patients never respond to their first biologic, and another 10 to 20% lose the response they had within a year [6,7]. Each failed drug means a fresh trial of the next one, and the interval between them carries real cost, both to the patient, whose joints keep deteriorating while inflammation runs unchecked, and to the health system paying for it.

In additions, this image would vary significantly if we knew beforehand who would respond to which medicine. unfortunately, the markers used today are not good enough. When evaluated honestly on external cohorts, anti-citrullinated protein antibodies, rheumatoid factor, disease activity scores seldom raise the AUC beyond 0.65, and 0.70 is the figure that most people consider the floor for a predictor worth utilising in the clinic [8]. So emphasis has shifted. One of the most intriguing avenues that search has explored is the gut microbiome.

There is good reason for the interest. The gut holds on the order of 10³ microbial cells and something like 33 million distinct microbial genes, and this community shapes the immune system in ways that reach far beyond the intestine. These include the induction and maintenance of Foxp3-expressing regulatory T cells, the restraint of pro-inflammatory Th17 lymphocytes, and the modulation of innate immune effector function in mucosal and systemic compartments [[9], [10], [11]]. Disturbance of this ecological equilibrium—broadly termed dysbiosis—is a consistent finding in RA and has been documented to precede the clinical onset of joint inflammation in prospective longitudinal cohorts [12,13]. Against this background, the present critical narrative review synthesises evidence linking pretreatment gut microbial composition to the magnitude of response to conventional and biologic DMARDs, appraises the pharmacomicrobiomic mechanisms through which the microbiome may actively shape drug action, and identifies the methodological barriers that must be surmounted before these findings can be translated into clinical practice.

## Search strategy and eligibility criteria

A structured, reproducible bibliographic search was performed across PubMed/MEDLINE, Scopus, Web of Science, the Cochrane Library, and the Directory of Open Access Journals, spanning January 2015 to April 2025. The start date was selected to capture literature published after the field-wide adoption of next-generation microbiome sequencing methods. The Boolean search string applied uniformly across databases was: ("gut microbiome" OR "gut microbiota" OR "intestinal microbiota") AND ("rheumatoid arthritis") AND ("treatment response" OR "biologic therapy" OR "predictive biomarker" OR "DMARD" OR "pharmacomicrobiomics" OR "methotrexate" OR "treatment outcome"). MeSH controlled vocabulary terms were incorporated in MEDLINE searches to maximise sensitivity.

**Fig. 1:**
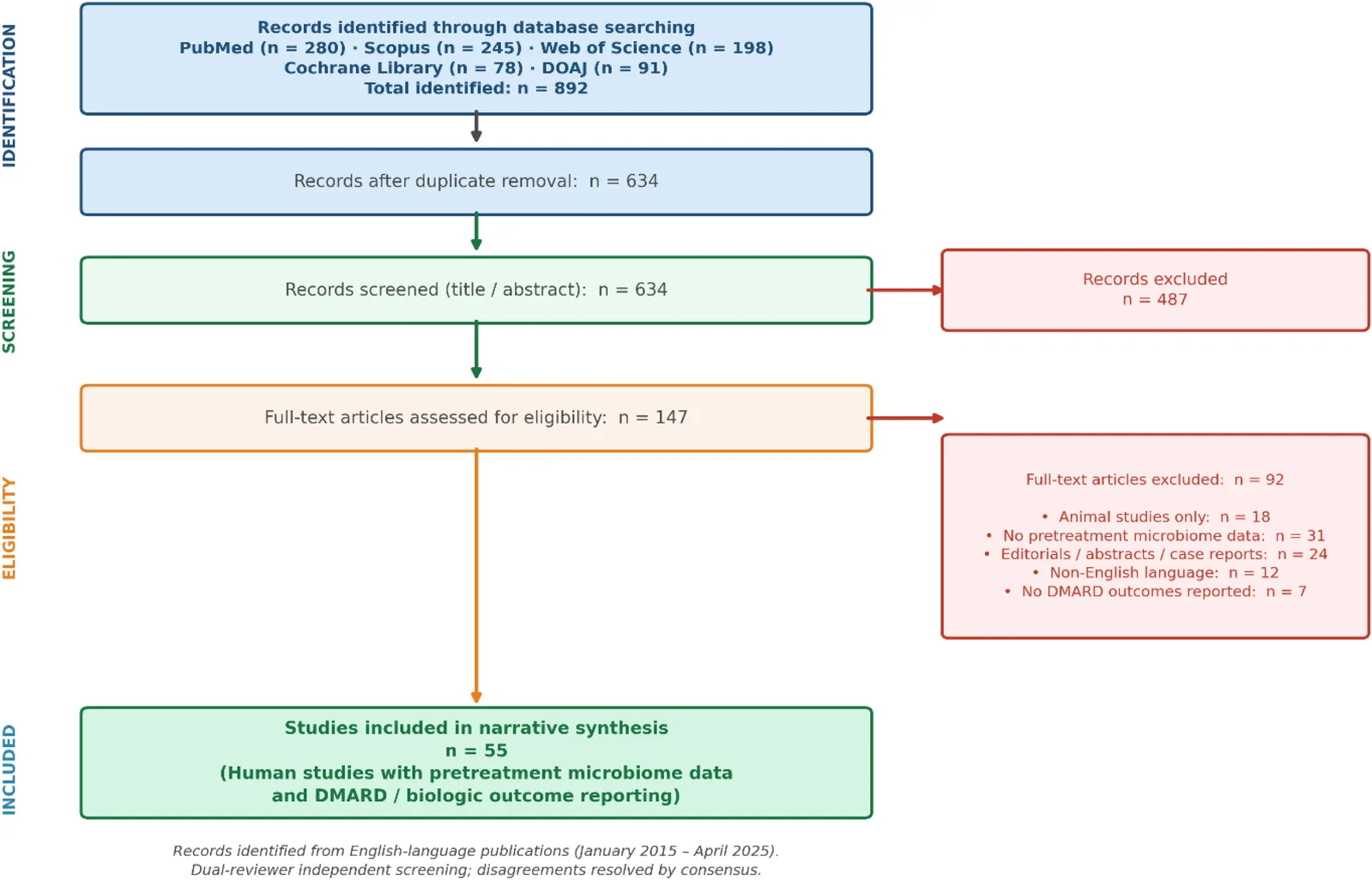
Presents a PRISMA-adapted search flowchart with verified stage-by-stage counts derived from the present review. Records were identified across five databases (PubMed n = 280, Scopus n = 245, Web of Science n = 198, Cochrane Library n = 78, DOAJ n = 91; total n = 892). After automated deduplication (n = 634 retained), dual-reviewer title/abstract screening excluded 487 records; full-text assessment of 147 articles led to exclusion of a further 92 (animal studies only n = 18; no pretreatment microbiome data n = 31; editorials/abstracts/case reports n = 24; non-English n = 12; no DMARD outcomes reported n = 7). Fifty-five studies satisfied all eligibility criteria and constitute the evidence base for this review.

Following automated removal of duplicates, two independent reviewers screened all titles and abstracts and subsequently assessed full texts of candidate records. Disagreements were resolved through discussion and consensus. Studies were eligible for inclusion if they: reported human data on pretreatment gut microbiome composition in relation to csDMARD or biologic DMARD outcomes; or examined pharmacomicrobiomic mechanisms pertaining to RA therapy. Exclusion criteria were: (1) animal-only experiments without human translational data; (2) failure to assess pretreatment microbiome composition or DMARD/biologic outcomes; (3) conference abstracts, letters, editorials, or case reports; or (4) non-English language of publication. Fifty-five articles satisfied these criteria and constitute the evidence base for this review. The included studies varied too much to combine statistically. They used different sequencing platforms, measured different outcomes, gave different drug regimens, and drew on cohorts that were not really comparable. Rather than force a meta-analysis onto data that could not support one, we synthesised the evidence narratively, following a consistent structure throughout (Fig. 1).

## Biologic therapies IN RA: efficacy and the predictive GAP

Five mechanistic classes of targeted therapy are now approved for RA: TNF inhibitors (etanercept, adalimumab, infliximab, certolizumab pegol, golimumab); IL-6 receptor antagonists (tocilizumab, sarilumab); the co-stimulation blocker abatacept; the B-cell depleter rituximab; and the JAK inhibitors tofacitinib, baricitinib, and upadacitinib, the last group classified as targeted synthetic DMARDs. TNF inhibitors remain the most frequently prescribed first-line biologics, delivering ACR20 response rates of 58–65% at 24 weeks in biologic-naïve cohorts [4]. IL-6 receptor blockade achieves comparable and in some settings superior results, with particular utility in seronegative patients and those with prominent systemic inflammation [14].

**Fig. 2:**
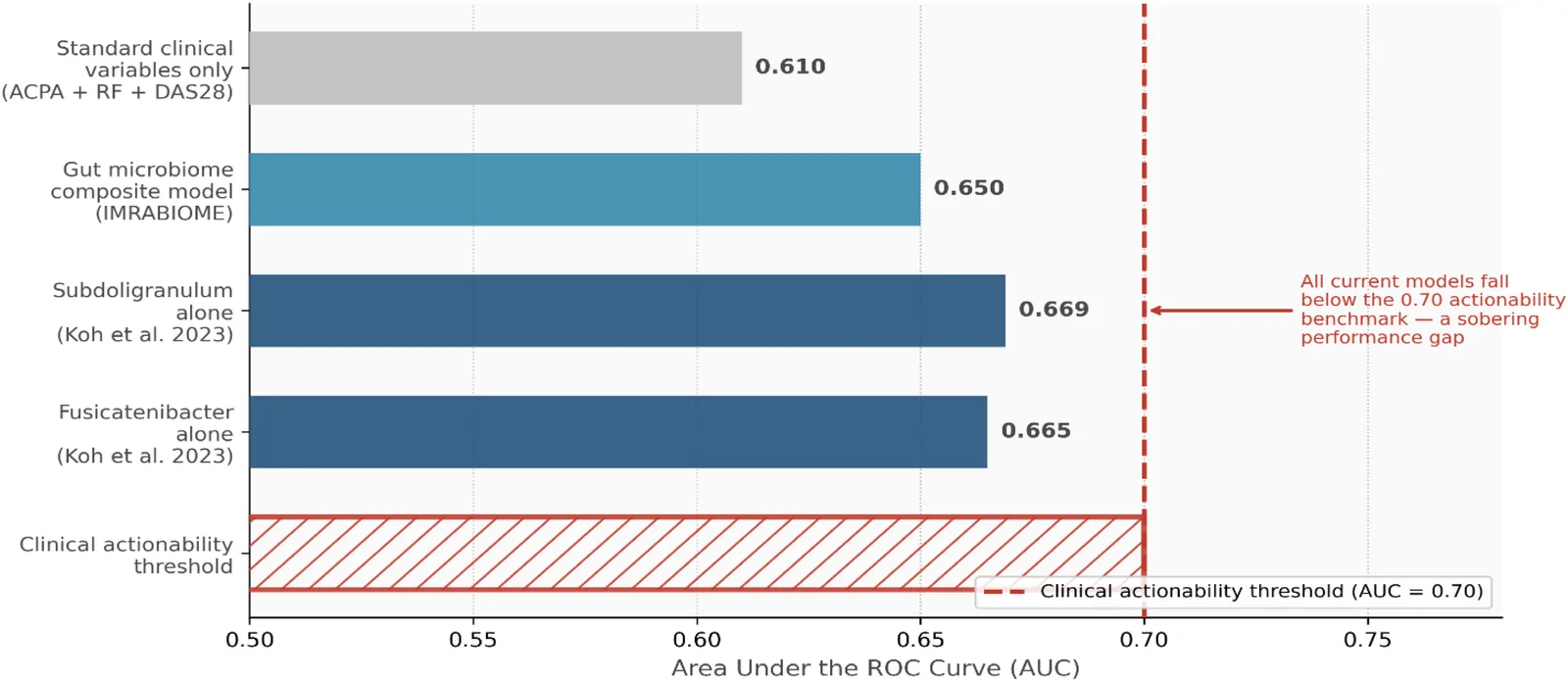
Comparison of predictive AUC values for gut microbiome-based versus standard clinical biomarker models for DMARD response in RA.

Despite this therapeutic breadth, the problem of non-response has proven difficult to resolve. Primary non-response—conventionally defined as failure to achieve ACR20 at 12 weeks—affects 30–40% of patients initiating first-line biologic treatment, and secondary non-response claims a further 10–20% annually [15]. Standard clinical and serological predictors exhibit AUC values below 0.65 in external validation, falling short of the 0.70 actionability benchmark established in the prediction modelling literature [8]. The message from these numbers is hard to miss: the biomarkers available now still cannot match treatment to patient with the precision the clinic needs. The microbiome models carry a similar warning. On external validation, IMRABIOME's composite model achieved an AUC of 0.65, only 0.04 higher than what routine clinical variables already provide, and both remain below the 0.70 generally needed for clinical action. This should be seen as a limitation rather than a green light for clinical use. Four hundredths of a point is unlikely to change anything at the bedside. Getting from here to a usable test that can inform real treatment decisions will require larger studies that combine microbial data with other kinds of information (Fig. 2).

## GUT microbiome dysbiosis IN rheumatoid arthritis

### Compositional alterations

**Fig. 3:**
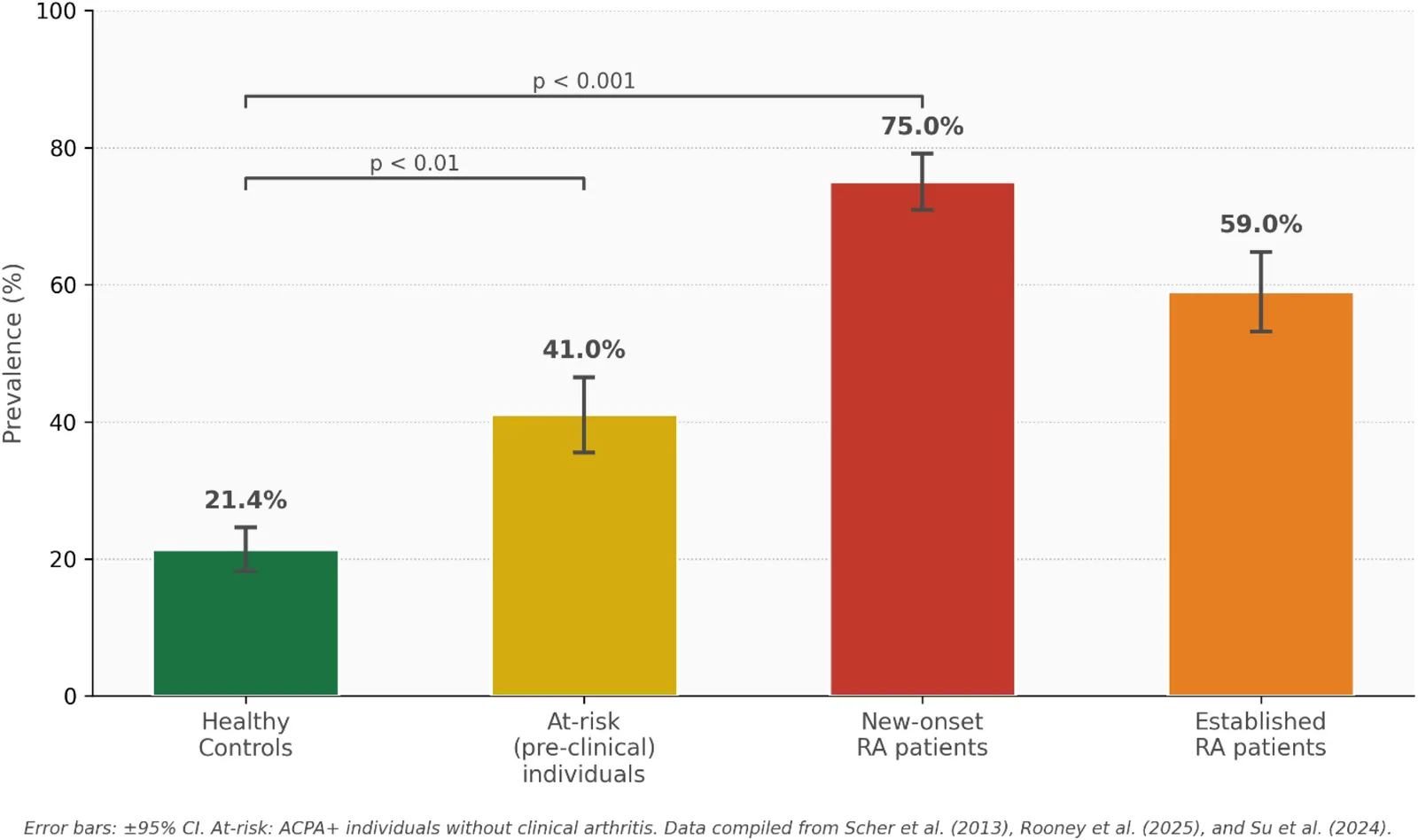
Prevotella copri prevalence in healthy controls, at-risk individuals, and patients with new-onset and established RA.

No microbial change in RA has been studied more than rise of Prevotella copri, a gram-negative anaerobic rod from the phylum Bacteroidota. The study differentiated between two groups, the first group to report it found the organism in 75% of newly diagnosed patients but only 21.4% of healthy controls (p < 0.001), and the finding has since been confirmed in cohorts from different regions and ethnic backgrounds [16]. Higher levels of P. copri are associated with higher ACPA titres and DAS28 scores. That happens before the disease develops is even more interesting. In people at high risk, the organism is already expanded in about 41% of cases before any signs of joint inflammation developed, placing it early in the chain of events leading to RA [[17], [18], [19]] (Fig. 3).

At the same time, this increase in pathobionts, the RA microbiome also shows a consistent loss of health-promoting commensal taxa. Faecalibacterium prausnitzii, the main butyrate-producing species in the healthy colon and one that accounts for 5–10% of total faecal biomass, is consistently under-represented in RA and inversely associated with disease activity across independent cohorts [20,21]. Bifidobacterium species are also reduced, and the degree of depletion is inversely correlated with DAS28-CRP composite scores (r = −0.42, p < 0.01) [22]. Members of the Lachnospiraceae family, particularly Subdoligranulum, Fusicatenibacter, and Roseburia hominis, are likewise depleted, with consequences for short-chain fatty acid production and Foxp3+ regulatory T-cell induction [23,24]. All these changes are illustrated in Table 1.

**Table 1:** Key gut microbial alterations in rheumatoid arthritis versus healthy controls.

Taxon	Direction in RA	Functional Role	Key Finding	Reference
*Prevotella copri*	↑ Increased	Pathobiont; encodes PAD-like citrullinating enzymes; potent IL-17 inducer	Detected in 75% of new-onset RA patients versus 21.4% of healthy controls (p < 0.001); titres correlate positively with ACPA and DAS28	Scher et al. eLife 2013 [16]
*Faecalibacterium prausnitzii*	↓ Decreased	Dominant colonic butyrate producer; inhibits NF-κB via HDAC suppression; anti-inflammatory	Consistently depleted across independent geographic cohorts; abundance inversely tracks disease activity indices	Sokol et al. PNAS 2008 [20]; He et al. Sci Adv 2022 [21]
Bifidobacterium spp.	↓ Decreased	SCFA synthesis; mucosal barrier maintenance; Treg expansion	Depletion magnitude inversely correlated with DAS28-CRP (r = −0.42, p < 0.01)	Wang et al. Front Microbiol 2022 [22]
*Subdoligranulum*	↓ Decreased	Butyrate-synthesising member of Lachnospiraceae	AUC = 0.669 for prediction of DAS28 ≤ 3.2 at six months in csDMARD-treated cohort	Koh et al. Arthritis Res Ther 2023 [23]
*Fusicatenibacter*	↓ Decreased	Butyrate-synthesising member of Lachnospiraceae	AUC = 0.665 for prediction of DAS28 ≤ 3.2 at six months in csDMARD-treated cohort	Koh et al. Arthritis Res Ther 2023 [23]
*Eggerthella lenta*	↑ Increased	Harbours cgr cytochrome-encoding gene cluster; participates in drug catabolism	Over-represented in RA microbiome profiles; implicated in inactivation of immunosuppressive compounds	Haiser et al. Science 2013 [25]
Lachnospiraceae (family)	↓ Decreased	Butyrate and propionate generation; supports Foxp3+ Treg pool	Family-level depletion correlates with elevated DAS28 and inferior treatment outcomes across multiple cohorts	Koh et al. 2023; Gupta et al. 2021 [23,26]
Shannon diversity index	↓ Reduced	Composite measure of species richness and evenness; proxy for ecosystem health	Pooled SMD = −0.61 (95% CI −0.89 to −0.33) in RA versus healthy controls across meta-analysed cohorts	Su et al. Explor Med 2024 [27]

ACPA, anti-citrullinated protein antibodies; AUC, area under the receiver operating characteristic curve; csDMARD, conventional synthetic disease-modifying antirheumatic drug; DAS28, Disease Activity Score in 28 joints; HDAC, histone deacetylase; PAD, peptidylarginine deiminase; SCFA, short-chain fatty acids; SMD, standardised mean difference; Treg, regulatory T cell..

### Diversity indices and disease activity

**Fig. 4:**
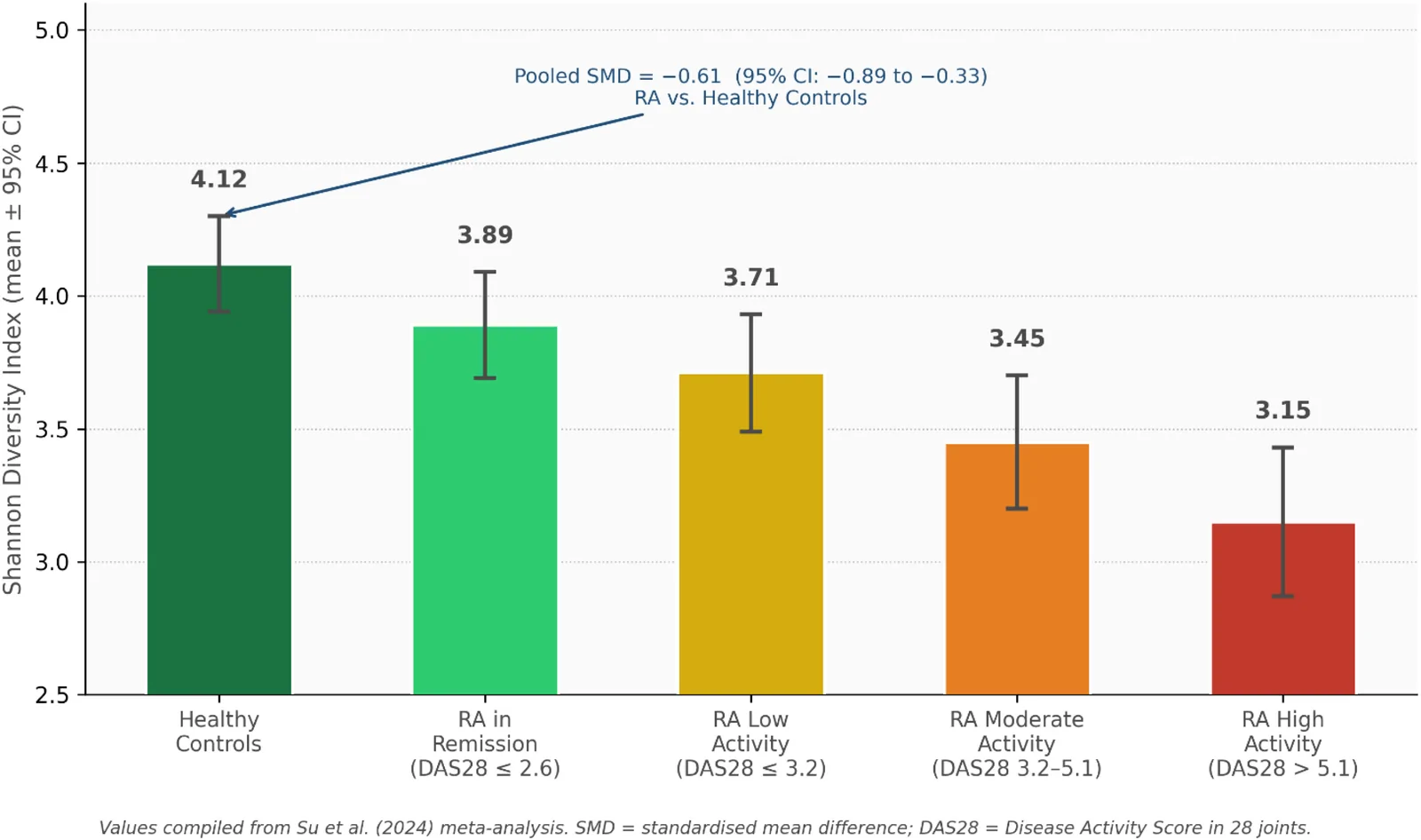
Shannon alpha-diversity with 95% confidence intervals across RA disease activity categories and healthy controls.

Alpha-diversity its measures the richness and evenness of a microbial community in a single sample. In RA, it's consistently lower than in healthy controls across independent cohorts. A meta-analysis combining several cohorts reported a standardized mean difference of −0.61 (95% CI: −0.89 to −0.33) for the Shannon index in RA compared with controls, indicating a meaningful reduction in diversity [27]. The same pattern also seen within the disease itself, where diversity declines as disease activity increases, reaching its lowest levels in patients with high disease activity (DAS28 > 5.1) [28]. The point worth keeping in mind is that the amount of diversity reported can vary depending on the sequencing platform and the bioinformatic pipeline used. This technical variation probably explains much of the disagreement in subgroup results across different studies [29] (Fig. 4).

## The gut–joint axis: mechanistic framework

**Fig. 5:**
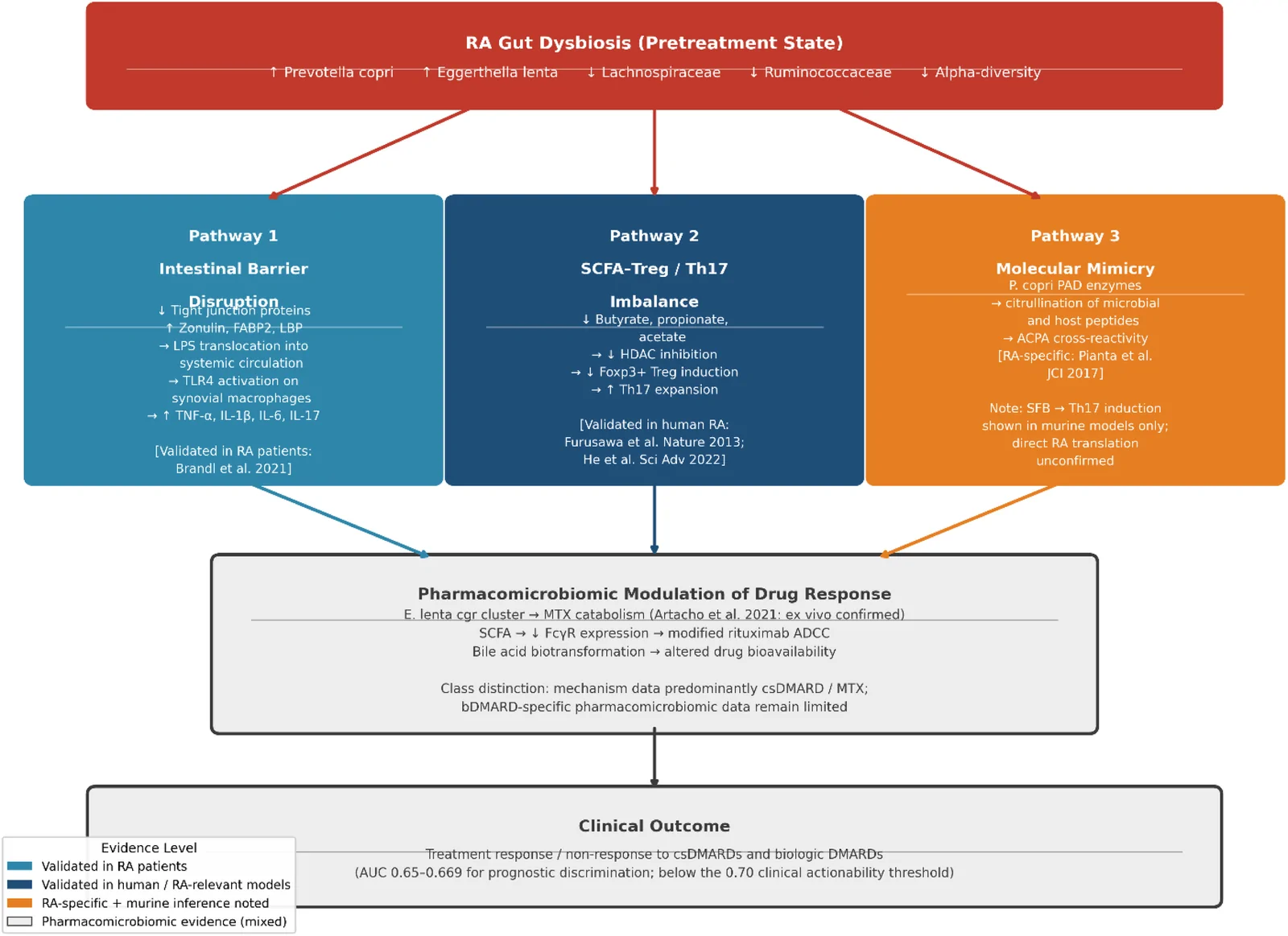
Schematic of the three mechanistic pathways by which intestinal dysbiosis drives synovial inflammation and modulates DMARD response in RA.

Three mechanisms link intestinal dysbiosis to joint inflammation in RA. They overlap in some ways, but each follows its own set of molecular mediators and has different therapeutic implications (Fig. 5).

The first involves a failing gut barrier. When the microbial community becomes disturbed, the tight junctions between intestinal cells lose their integrity, and this is reflected in the serum, where zonulin, fatty acid-binding protein 2, and lipopolysaccharide-binding protein all increase [30]. As a result, gram-negative bacterial lipopolysaccharides can translocate into the systemic circulation and activate Toll-like receptor 4 signalling on synovial macrophages and dendritic cells. This drives the production of TNF-alpha, IL-1 beta, IL-6, and IL-17, which are the main cytokines targeted by the biologic drugs currently used to treat RA [30,31]. The second pathway centres on the depletion of butyrate-producing taxa within the Lachnospiraceae and Ruminococcaceae families. Reduced intestinal concentrations of butyrate, propionate, and acetate impair histone deacetylase inhibition and attenuate Foxp3 acetylation, thereby compromising the generation and stability of Foxp3-expressing regulatory T cells. The resulting immunological imbalance, characterised by diminished Treg activity and expanded Th17 populations, promotes the pro-inflammatory joint environment that is the hallmark of active RA [32].

Peptidylarginine deiminase (PAD)-like enzymes expressed by P. copri citrullinate both microbial and host peptides, generating neoantigens structurally similar to the citrullinated self-epitopes recognised by RA-specific anti-citrullinated protein antibodies. In RA patients specifically, Pianta et al. (2017) demonstrated T-cell cross-reactivity between P. copri-derived antigens and synovial autoantigens, providing direct RA-patient evidence that PAD-mediated citrullination may contribute to ACPA generation [33]. It should be emphasised, however, that molecular mimicry represents one of several non-mutually exclusive pathogenic mechanisms: bystander activation and epitope spreading also contribute to the perpetuation of synovial autoimmunity and cannot be excluded on the basis of current cross-sectional evidence. The induction of intestinal Th17 cells by segmented filamentous bacteria, while well-established in murine models, has not been directly validated in human RA cohorts and should be regarded as a biologically plausible inference rather than an established RA-specific mechanism.

## Baseline gut microbiome as a predictor of treatment response

### Key clinical studies

Danckert et al. (2024) carried out the largest prospective investigation of pretreatment microbiome and DMARD response conducted to date—the IMRABIOME study—enrolling 144 treatment-naïve early RA patients across 12 National Health Service rheumatology centres in the United Kingdom [34]. Shotgun whole-metagenome sequencing was performed on faecal and saliva samples obtained at treatment initiation, six weeks, and twelve weeks. Pretreatment gut microbiota composition predicted achievement of minimum clinically important improvement at six weeks, and a sparse partial least squares discriminant analysis model of microbial features achieved AUC = 0.65 in a geographically separate validation cohort—the first robust external validation of a microbiome-based response prediction model in RA. Thirteen bacterial taxa declined specifically in clinical responders by six weeks, of which seven were Prevotella species. Partial microbiome restoration toward a eubiotic pattern was observed exclusively in responders, consistent with a bidirectional relationship between treatment efficacy and microbial ecology. Critical Commentary: Despite its strengths as the largest prospective study in this field, IMRABIOME has notable limitations. The patients all came from a single national health service and were overwhelmingly of White European descent, so how far the results extend to other populations is uncertain. There is also the matter of treatment: rather than a single standardised protocol, patients received a mix of csDMARD regimens, and that variation introduces confounding the available covariates cannot fully account for. The AUC of 0.65 in external validation, while the highest composite value in this literature, falls below the 0.70 clinical actionability threshold and should not be interpreted as evidence that microbiome profiling is ready for clinical deployment. Risk of bias is moderate: no pre-specified biomarker threshold was defined before unblinding, raising the possibility of implicit optimisation toward observed outcomes.

Koh et al. (2023) conducted a prospective Korean cohort study (n = 94 RA patients, 30 healthy controls) using 16S rRNA V3–V4 amplicon sequencing processed through the QIIME2 pipeline [23]. Treatment response was defined as attainment of DAS28 ≤ 3.2 at six-month follow-up. Pretreatment abundance of Subdoligranulum and Fusicatenibacter individually achieved AUC = 0.669 and AUC = 0.665, respectively—the highest single-taxon AUC values documented for microbiome biomarkers of DMARD response in RA in the published literature as of this review. Both taxa belong to the butyrate-producing Lachnospiraceae and are mechanistically consistent with the SCFA–Treg regulatory axis described in Section 5.

Critical Commentary: The Koh et al. study reports the highest single-taxon AUC values in this literature (Subdoligranulum 0.669; Fusicatenibacter 0.665). Several limitations temper their interpretation. First, both values derive from an internal cohort without independent external validation, raising the possibility of optimistic bias. Second, the single-country Korean population limits cross-ethnic generalisability, particularly given the documented geographic variability of gut microbial ecology. Third, single-taxon AUCs of 0.665–0.669 remain below the 0.70 actionability threshold and are unlikely to support clinical decision-making in isolation. These findings are best regarded as internally consistent hypothesis-generating signals requiring prospective external replication.

Artacho et al. (2021) conducted the first dedicated pharmacomicrobiomic study of treatment response in drug-naïve new-onset RA, with a training cohort of 26 patients and an independent validation cohort of 21 patients [35]. Pretreatment shotgun metagenomic sequencing was performed within 48 h of initiating methotrexate therapy. Machine-learning analysis of metagenomic functional data identified bacterial orthologs involved in purine biosynthesis and MTX catabolism as the most discriminating features separating subsequent responders from non-responders. Ex vivo incubation experiments provided direct experimental evidence that pretreatment faecal microbiota catabolise methotrexate; the residual drug concentration after faecal incubation correlated significantly with clinical response magnitude. These pharmacomicrobiomic findings indicate that baseline microbiome composition may influence not only biomarker prediction but also actual drug bioavailability and, by extension, therapeutic efficacy. Critical Commentary: Artacho et al. stands alone as the only published study to show, through direct ex vivo experiments, that gut bacteria catabolise a drug in the RA setting, and that experimental evidence gives its mechanistic argument real weight. The sample sizes are the weakness. With 26 patients in the training set and 21 in the validation set, the study is too small to identify biomarkers reliably once the analysis is corrected for multiple testing. The study design is observational and cannot confirm that microbial MTX catabolism causally reduces clinical efficacy; drug-disease interactions, patient adherence, and pharmacokinetic variability are not fully modelled. Internal validity is limited by the absence of an independent replication cohort with sufficient size. These findings are best regarded as hypothesis-generating mechanistic data requiring confirmation in prospective cohorts of at least 200 patients.

Gupta et al. (2021) performed a longitudinal investigation of 32 RA patients with faecal samples collected at two time-points (64 samples total) using shotgun whole-metagenome sequencing at the Mayo Clinic, USA [26]. l-arginine biosynthesis and butyrate metabolism pathways were significantly enriched among patients who achieved minimum clinically important improvement on the Clinical Disease Activity Index, offering functional metabolic resolution that complements taxon-level observations. Zhang et al. (2015), in a foundational cohort study published in Nature Medicine (n = 77; China), demonstrated that both the oral and gut microbiomes are substantially perturbed in RA and undergo partial normalisation in response to DMARD therapy, with the extent of microbiome recovery correlating inversely with subsequent DAS28 change (r = −0.38, p = 0.004) [36]. These five core studies are complemented by cohort-level investigations by Jeong et al. (2019) and Rooney et al. (2025), which are summarised alongside them in Table 2.

**Table 2:** Summary of key studies linking pretreatment gut microbiome composition to RA treatment response.

Study (Year)	n	Design / Setting	Sequencing	Treatment	Key Finding	Effect Size
Danckert et al. Rheumatology 2024 [34]	144 (DMARD-naïve)	Prospective longitudinal; 12 UK rheumatology centres (IMRABIOME)	Shotgun WMS baseline, 6 wk, 12 wk	Mixed csDMARDs	Baseline gut microbiome composition predicted MCII at six weeks. Thirteen taxa fell in responders, seven of which were Prevotella species. Partial microbiome restoration toward eubiosis was exclusive to responders.	AUC = 0.65 (validation cohort)
Koh et al. Arthritis Res Ther 2023 [23]	94 RA; 30 HC	Prospective cohort; Catholic University, South Korea	16S rRNA V3–V4; QIIME2 pipeline	Second-line csDMARDs (DAS28 ≥ 3.2)	Subdoligranulum and Fusicatenibacter baseline abundance predicted DAS28 ≤ 3.2 at six months. Both are butyrate-producing Lachnospiraceae, consistent with the SCFA–Treg regulatory pathway.	AUC = 0.669 (Subdoligranulum) AUC = 0.665 (Fusicatenibacter)
Artacho et al. Arthritis Rheumatol 2021 [35]	26 training; 21 validation	Pharmacomicrobiomic prospective; drug-naïve new-onset RA	Shotgun WMS within 48 h of treatment start	Methotrexate (first-line)	Machine-learning models identified purine biosynthesis and MTX-metabolism bacterial orthologs as key discriminators of subsequent response. Ex vivo faecal incubation confirmed direct microbial MTX catabolism; residual drug levels correlated with clinical outcome.	Feature importance (ML model); r with response (ex vivo)
Gupta et al. Genome Med 2021 [26]	32 (64 samples, 2 time-points)	Longitudinal; Mayo Clinic, USA	Shotgun WMS	Mixed DMARDs	L-arginine biosynthesis and butyrate metabolism pathways enriched in CDAI-MCII achievers, providing functional metabolic depth beyond taxon-level inference.	Pathway enrichment association (no single AUC)
Zhang et al. Nat Med 2015 [36]	77	Cohort; China	Shotgun WMS	Conventional and biologic DMARDs	Oral and gut microbiome composition was substantially perturbed in RA and underwent partial normalisation after DMARD therapy; recovery magnitude correlated inversely with subsequent DAS28 change.	r = −0.38, p = 0.004 (microbiome change vs. DAS28 change)
Jeong et al. J Clin Med 2019 [28]	33 early RA; 30 HC	Cross-sectional; South Korea	16S rRNA V3–V4	CsDMARDs (early RA)	Distinct microbial profiles were observed between early RA and healthy controls. Lachnospiraceae and Ruminococcaceae were reduced. Alpha-diversity correlated negatively with DAS28 scores.	Alpha-diversity inversely correlated with DAS28
Rooney et al. Ann Rheum Dis 2025 [29]	Pre-clinical cohort	Longitudinal observational; at-risk individuals; UK	16S rRNA; longitudinal sampling	None (pre-clinical)	Gut microbial dynamics were characterised in individuals at risk of RA prior to clinical diagnosis. Dysbiosis antedated arthritis onset, supporting potential utility as an early biomarker.	Descriptive; cohort dynamics

AUC = area under the receiver operating characteristic curve; CDAI = Clinical Disease Activity Index; csDMARD = conventional synthetic disease-*modifying antirheumatic drug; DAS28 = Disease Activity Score in 28 joints; HC = healthy controls; MCII = minimum clinically important improvement; ML = machine learning; MTX = methotrexate; SCFA = short-chain fatty acids; Treg = regulatory T cell; WMS = whole metagenome sequencing.*

Critical Commentary: Zhang et al. (2015) is a foundational study demonstrating that the oral and gut microbiomes are perturbed in RA and partially normalise after DMARD therapy. Its principal limitation for the present review is that it characterises treatment-associated microbiome change rather than pretreatment prediction; the reported inverse correlation between microbiome recovery and DAS28 change (r = −0.38) reflects a prognostic association on fixed therapy, not a treatment-selection signal. The cross-sectional nature of the comparative analyses means that causality cannot be established, and the lack of a validation cohort reduces confidence in the specific taxa identified. For this reason, the study is better cited as mechanistic and descriptive context rather than as direct evidence of pretreatment prognostic capacity.

Critical commentary: The study by Gupta et al. provides useful functional metagenomic insight, identifying enriched l-arginine biosynthesis and butyrate metabolism pathways in treatment responders. However, the cohort of 32 patients, even with 64 serial samples, is substantially underpowered for reliable biomarker discovery after correction for the high dimensionality of metagenomic pathway data. Recruitment from a single tertiary referral centre (Mayo Clinic) may introduce selection bias toward more severe or refractory disease relative to community RA populations. No single-taxon or composite AUC was reported, precluding direct comparison with prognostic discrimination metrics from other studies. The pathway-level findings are mechanistically informative but require confirmation in adequately powered independent cohorts.

Interpretive note on prediction model performance: Across the studies evaluated, microbiome-based prediction models showed marginally higher AUC values than standard clinical biomarker models. However, this apparent advantage (AUC 0.65 vs. 0.61; delta = 0.04) must not be framed as clinical superiority: both models fall well below the 0.70 actionability threshold, and a delta of 0.04 confers no meaningful advantage in clinical decision-making. However, the highest validated composite AUC—0.65 in the IMRABIOME external validation cohort—remains below the 0.70 benchmark widely regarded as the minimum for clinical decision support. This performance shortfall must be explicitly acknowledged and addressed through adequately powered multicenter validation before microbiome profiling can be recommended as a clinical tool for DMARD selection.

### Pharmacomicrobiomics: microbial modulation of drug pharmacodynamics

The pharmacomicrobiomic paradigm extends the role of the gut microbiome from a predictor of treatment response to an active modulator of drug pharmacokinetics and pharmacodynamics. Short-chain fatty acids produced by commensal bacteria inhibit histone deacetylases and regulate the expression of Fc-gamma receptors on natural killer cells and macrophages. This mechanism that may affect the antibody-dependent cellular cytotoxicity that contribute the efficacy of rituximab in RA [24,37]. there is another example, Eggerthella lenta, a gram-positive anaerobe enriched in some RA-associated microbiome profiles, encodes the cgr cytochrome gene cluster, which mediates the catabolism of a range of immunosuppressive xenobiotics [25]. In addition, pharmacomicrobiomic pathways such as bile acid biotransformation and tryptophan catabolism, may also have downstream effects on systemic immune regulation and drug response [[37], [38], [39]]. overall, this findings supports the view that the gut microbiome is an active pharmacological participant rather than simply a passive correlate of treatment outcome.

## Methodological challenges and sources of heterogeneity

### Technical variability in microbiome profiling

The biggest common technical challenge in comparing microbiome studies is the lack of a common methodological standard for microbiome profiling. Targeted 16S rRNA amplicon sequencing and shotgun whole-metagenome sequencing agree on genus-level assignments in only about 68% of cases, meaning that almost one-third of detected genera may reflect platform-specific differences rather than true biological variation [40,41]. ThThe problem is made even more complex by differences in target hypervariable regions (V3–V4 versus V1–V3), sequencing depth, DNA extraction methods, and bioinformatic pipelines such as QIIME2, MetaPhlAn4, and Kraken2 [42]. As a result, these technical differences make it difficult to identify biomarker taxa that are reproduced across independent studies, even when the patient populations and clinical questions are very similar.

### Study design and statistical limitations

Most published RA microbiome studies have the same design features that limit how their findings can be interpreted. Typical cohort sizes usully range from 30 to 60 participants per each group, which is generally not enough to detect the modest effect sizes associated with individual microbial taxa after appropriate multiple-testing correction. Furthermore, Most studies are cross-sectional or retrospective, making it defficult to draw the conclusions about causality. Another chalnges is the lack of harmonised response definitions, with ACR20, ACR50, DAS28-remission, CDAI, and MCII often used interchangeably, making it difficult to combine response data across studies in a statistically valid way. Methotrexate, the anchor csDMARD in RA treatment, also affects gut microbiome composition through its direct antimicrobial activity, independent of its immunosuppressive effects, making it an important confounding factor that most published studies do not adequately control for [35,43]. Proton pump inhibitors have a similar issue, which they are commonly prescribed alongside NSAIDs, have measurable and reproducible effects on intestinal Streptococcus and Clostridiales populations but are rarely included as analytic covariates [44].

### Ethnic and geographic underrepresentation

A clear geographic imbalance exists in the RA microbiome literature. Most of the available published studies come from East Asian populations, mainly Chinese and Korean, while patients from the Middle East, sub-Saharan Africa, and South Asia are largely missing from the current evidence base. This gap is important because the composition of the gut microbiome is shaped by factors that differ across regions, including habitual diet, host genetic background, environmental exposures, and patterns of antibiotic use [45,46]. A biomarker developed and validated exclusively in East Asian populations may perform differently in Middle Eastern, African, or South Asian patients a critical consideration for a disease that affects individuals worldwide. The methodological challenges identified above and their proposed mitigations are presented systematically in Table 3.

**Table 3:** Principal methodological challenges in RA gut microbiome research and proposed mitigation strategies.

Challenge	Description	Impact on Study Findings	Proposed Mitigation
Sequencing platform heterogeneity	16S rRNA amplicon versus shotgun whole-metagenome sequencing; differing primer sets and variable read depth	Cross-platform genus-level concordance of approximately 68%, limiting quantitative comparison across studies	Mandatory adoption of shared bioinformatic pipelines (QIIME2, MetaPhlAn4); parallel-platform profiling in discovery cohorts
Insufficient sample sizes	Median group size of 30–60 participants in published RA microbiome studies	Underpowered detection of modest effect sizes; elevated false-discovery rates; restricted generalisability	Multicenter consortium designs; minimum n ≥ 300 per analytical group in validation phases
csDMARD confounding	Methotrexate exerts intrinsic antimicrobial activity on the intestinal flora, independent of disease suppression	Baseline microbiome–response associations are confounded in mixed-treatment cohorts without stratification	Pre-specified treatment stratification; dedicated biologic-naïve and DMARD-naïve subgroup analyses
Dietary confounding	Habitual diet explains 15–20% of gut microbiome variance; nutritional data are rarely collected systematically	Spurious taxon–outcome associations attributable to unmeasured dietary differences between groups	Validated food-frequency questionnaires; Mediterranean Diet Adherence Score as a pre-specified covariate
Proton pump inhibitor and NSAID use	PPIs significantly alter Streptococcus and Clostridiales abundances; frequently prescribed in RA alongside NSAIDs	Uncontrolled confounding in the majority of published RA microbiome investigations	Pre-specified PPI covariate adjustment; PPI use as a formal stratification variable
Outcome measure heterogeneity	ACR20, ACR50, DAS28, CDAI, and MCII employed interchangeably across studies without pre-registration	Valid meta-analytic pooling of response data is precluded	OMERACT-endorsed consensus on standardised outcome reporting for microbiome–treatment response studies
Geographic and ethnic underrepresentation	East Asian cohorts dominate the evidence base; Middle Eastern, African, and South Asian populations are absent	Generalisability is constrained; geographic microbiome variation may distort biomarker performance estimates	International multicenter designs with mandatory ethnic stratification and subgroup reporting
Pre-analytical sample variability	Heterogeneous collection media, DNA extraction kits, and freeze–thaw protocols across laboratories	Technical variation may exceed true biological signal differences	Standardised OMNIgene·GUT collection kits; centralised DNA extraction and biobanking

ACR = American College of Rheumatology; CDAI = Clinical Disease Activity Index; csDMARD = conventional synthetic disease-modifying antirheumatic drug; DAS28 = Disease Activity Score in 28 joints; MCII = minimum clinically important improvement; NSAID = non-steroidal anti-inflammatory drug; OMERACT = Outcome Measures in Rheumatology; PPI = proton pump inhibitor; WMS = whole metagenome sequencing.

## Clinical implications and future directions

### Toward microbiome-guided treatment selection

While the current body of microbiome-treatment response evidence does not yet meet the evidentiary standards required for direct clinical adoption, it constitutes an internally consistent proof-of-principle that intestinal microbial profiling carries prognostic value for DMARD and biologic drug response. A clinically deployable microbiome-based biomarker panel would need to centre on a tractable set of quantifiable taxa, detectable by cost-efficient 16S rRNA amplicon sequencing or a validated qPCR panel, and accompanied by alpha-diversity indices and surrogate measures of butyrate biosynthetic capacity. Prediction models integrating microbiome features with standard clinical variables would require validated AUC ≥ 0.70 in prospective external validation before entering clinical decision-support pathways. Among the computational approaches evaluated to date, ensemble machine-learning frameworks—including random forest classifiers, gradient-boosted decision trees, and support vector machines—have demonstrated the most consistent capacity to exploit high-dimensional microbial datasets alongside heterogeneous clinical covariates [48,49].

### Microbiome modulation as a therapeutic strategy

Beyond its potential as a predictor of treatment response, the gut microbiome is also a modifiable therapeutic target that may improve the effectiveness of pharmacological treatments through pharmacomicrobiomic mechanisms [50,51]. In pilot RA cohorts, high-fibre dietary regimens that promote the expansion of butyrate-producing Lachnospiraceae taxa while reducing P. copri colonisation have been associated with measurable reductions in circulating inflammatory markers [52]. Probiotic supplementation with *Lactobacillus acidophilus*, Bifidobacterium bifidum, and Bacillus coagulans has yielded statistically significant, albeit modest, reductions in DAS28 scores and pro-inflammatory cytokine concentrations in randomised trials; a recent meta-analysis encompassing 80 RCTs across 14 autoimmune conditions provided pooled evidence supporting these effects [55]. Faecal microbiota transplantation remains in a pre-trial investigational phase for RA—no published randomised controlled trial data were available as of April 2025—but the theoretical underpinning for transferring a eubiotic donor microbiome as an adjunct to biologic DMARD therapy is scientifically coherent and warrants dedicated prospective evaluation [53,54].

### Priority research agenda

On the basis of the evidence reviewed, the following priorities warrant immediate investigative attention. Multicenter prospective cohort studies should enrol at minimum 300 biologic-naïve RA patients per cohort, with standardised faecal sampling at treatment initiation, harmonised whole-metagenome sequencing using a shared bioinformatic pipeline, and pre-registered clinical outcome assessment at 12 and 24 weeks. International expert consensus is needed to define minimum reporting standards for RA microbiome-treatment response studies, analogous to the STROBE-MR framework for Mendelian randomisation. Integration of functional metagenomics with metabolomic profiling at pretreatment baseline will be necessary to fully characterise the short-chain fatty acid biosynthetic capacity of individual patients. Standardised dietary assessment using validated instruments—specifically validated food-frequency questionnaires and the Mediterranean Diet Adherence Score—must be incorporated as a mandatory covariate in all future predictive modelling studies, rather than treated merely as a confounder to control. Habitual diet accounts for 15–20% of gut microbiome variance [47] and represents one of the most powerful modifiable determinants of microbial ecology; its systematic collection and integration into prediction models may substantially improve discriminative accuracy and is considered a priority research gap by this review. Multi-omic models combining microbial, genomic, transcriptomic, and metabolomic features represent the most promising route toward composite predictive biomarkers with sufficient discriminative accuracy for clinical deployment. Finally, prospective cohort studies must systematically include patients from Middle Eastern, African, and South Asian populations to establish the cross-ethnic generalisability of microbiome-based biomarker findings.

## Conclusion

A substantial and methodologically diverse body of evidence now supports the proposition that pretreatment gut microbiome composition encodes clinically relevant prognostic information about the magnitude of response to both conventional synthetic and biologic DMARDs in treatment-naïve rheumatoid arthritis patients. The reproducible association between higher baseline abundance of butyrate-producing Lachnospiraceae taxa—particularly Subdoligranulum (AUC = 0.669) and Fusicatenibacter (AUC = 0.665)—and favourable csDMARD outcomes at six months, together with composite microbiome models achieving AUC = 0.65 in independent external validation, represents the strongest available quantitative evidence in this field to date.

The biological coherence of these associations is supported by four distinct but interconnected mechanisms: butyrate-mediated regulation of the Treg/Th17 balance, direct pharmacomicrobiomic modulation of drug bioavailability, maintenance of intestinal barrier integrity, and molecular mimicry between microbial citrullinated antigens and synovial autoantigens. Particularly important are the ex vivo pharmacomicrobiomic findings showing direct methotrexate catabolism by pretreatment faecal microbiota, which move the microbiome beyond a correlational biomarker and support its role as a potential causal mediator of treatment outcome.

It is equally important to acknowledge the present limitations with candour. Even the highest validated composite AUC in this literature—0.65 in the IMRABIOME external cohort—remains meaningfully below the 0.70 minimum required for clinical decision support, a threshold that itself represents a floor rather than a ceiling for actionable biomarkers. Future studies must not only raise AUC values above 0.75 but also demonstrate, through prospective randomised designs, that microbiome-guided treatment decisions improve patient-relevant outcomes—including avoidance of ineffective therapy, reduction of disease progression during drug-failure intervals, and decreased healthcare expenditure—before microbiome profiling can be incorporated into clinical rheumatological guidelines. Neither the consistency nor the generalisability of microbiome-based prognostication has been established across adequately powered, ethnically diverse prospective cohorts. Technical heterogeneity in sequencing platforms and bioinformatic pipelines, small and geographically restricted study populations, inadequate control of key confounders including methotrexate and proton pump inhibitors, and non-standardised outcome definitions all limit the conclusions that can currently be drawn. Resolving these limitations will necessitate coordinated international multicenter efforts, methodological harmonisation, and the systematic integration of multi-omic data streams. Only by addressing these foundational gaps will the field be positioned to translate its preliminary findings into reliable, clinically deployable precision prescribing tools for RA management.

## Ethical statement and AI use disclosure

This narrative review did not involve primary data collection, experimental procedures in human subjects, or animal research; accordingly, no institutional ethics committee approval was sought or required. All primary studies cited were previously reviewed and approved by appropriate ethics bodies as documented in their respective original publications.

Artificial intelligence writing-assistance tools were employed during manuscript preparation exclusively for editorial refinement—specifically grammar checking, spelling correction, and phrasing clarity. All substantive intellectual content, including the analytical framework, evidence synthesis, critical appraisal, mechanistic interpretations, and conclusions, was formulated independently by the named authors. Every section of the manuscript was critically reviewed, substantively revised, and approved by all authors before submission. AI model consulted for editorial assistance: Claude (Anthropic). No section of this manuscript was submitted in AI-generated form without comprehensive independent scholarly review and revision.

## Disclaimer

No portion of this manuscript has been reproduced from previously published works, submitted concurrently to any other journal, or produced in its submitted form by an artificial intelligence system acting without independent scholarly review and revision by the named authors. All literature cited was independently accessed and verified by the authorship team.

## Financial disclosures

This research received no specific grant from any funding agency in the public, commercial, or not-for-profit sectors. No pharmaceutical company, biotechnology firm, diagnostic company, or other commercial entity provided financial support, materials, or any form of assistance for this work.

## Non-financial disclosures

No author holds advisory board positions, consultancy roles, speaker bureau memberships, or patent interests related to gut microbiome diagnostics, DMARD therapy, or rheumatological precision medicine. No author has received honoraria, travel grants, or other compensation from entities with a financial interest in the subject matter of this manuscript.

## Open access funding

This research received no specific funding from any public, commercial, or not-for-profit funding agency. Where open access publication charges apply, the corresponding author will seek institutional support from Mustansiriyah University, Baghdad, Iraq. No external commercial manuscript editing or language-polishing services were retained for this work.

## CRediT authorship contribution statement

**Ali Saad Al-Budairi:** Writing – original draft, Funding acquisition, Formal analysis, Data curation, Conceptualization. **Abdullah Salim Al-Karawi:** Writing – review & editing, Supervision, Software, Resources. **Ali M. Atoom:** Visualization, Validation, Supervision.

## Declaration of competing interest

The authors declare that they have no known competing financial interests or personal relationships that could have appeared to influence the work reported in this paper.

## Data Availability

Data will be made available on request.
